# Masculinized Sexual Ornaments in Female Lizards Correlate with Ornament-Enhancing Thermoregulatory Behavior

**DOI:** 10.1093/iob/obac029

**Published:** 2022-08-25

**Authors:** B A Assis, J D Avery, R L Earley, T Langkilde

**Affiliations:** Department of Biology, Intercollege Graduate Degree Program in Ecology, The Pennsylvania State University, University Park, PA 16802; Department of Ecosystem Science and Management, Intercollege Graduate Degree Program in Ecology, Pennsylvania State University, University Park, PA 16802; Department of Biological Sciences, University of Alabama, Tuscaloosa, AL 35487; Department of Biology, Intercollege Graduate Degree Program in Ecology, The Pennsylvania State University, University Park, PA 16802

## Abstract

The adaptive significance of colorful or exaggerated traits (i.e., ornaments) expressed in females is often unclear. Competing hypotheses suggest that expression of female ornaments arises from maladaptive (or neutral) genetic inheritance from males along with incomplete epigenetic regulation, or from positive selection for ornaments in females under social competition. Whether costly or advantageous, the visibility of such traits can sometimes be behaviorally modulated in order to maximize fitness. Female eastern fence lizards express blue badges that are variable in size and color saturation. These are rudimentary compared to those seen in males and carry important costs such as reduced mating opportunities. Body temperature is a well-established enhancer of badge color, and thus thermoregulation may be one way these animals modulate badge visibility. We quantified realized body temperatures of female lizards paired in laboratory trials and observed that females with larger badges attained higher body temperatures when freely allowed to thermoregulate, sometimes beyond physiological optima. In this association between phenotype and behavior, females with larger badges exhibited thermoregulatory patterns that increase their badges’ visibility. This signal-enhancing behavior is difficult to reconcile with the widely held view that female ornaments are maladaptive, suggesting they may carry context-dependent social benefits.

## Portuguese

É muitas vezes incerta a significância adaptativa de caracteres vívidos e coloridos em fêmeas. Hipóteses para esse fenômeno sugerem uma herança maladaptativa (ou neutra) de caracteres selecionados em machos aliada à regulação epigenética incompleta em fêmeas, ou ainda seleção positiva em fêmeas imposta por competição social. Vantajosos ou não, a visibilidade de ornamentos muitas vezes é modulada por vias comportamentais do portador de modo a balancear seus custos e benefícios. Fêmeas *Sceloporus undulatus* possuem um par de marcas verde-azuis na parte ventral do pescoço que são variáveis em área e saturação entre indivíduos. Esses ornamentos são rudimentares em relação aos vistos em machos, mas ainda assim estão associados a custos reprodutivos importantes. Nessa espécie, temperatura corporal aumenta a visibilidade desses ornamentos significativamente. Portanto, a termorregulação é um comportamento que fêmeas poderiam empregar para modular a visibilidade de ornamentos. Nós quantificamos temperaturas corporais obtidas por pares de lagartos fêmeas em testes comportamentais e observamos que fêmeas com os maiores ornamentos também obtiveram temperaturas corporais mais altas, às vezes além do ótimo fisiológico. Nesta associação entre fenótipo e comportamento, fêmeas com os maiores ornamentos exibiram padrões de termoregulação que aumentaram sua visibilidade. Este padrão é difícil de conciliar com a perspectiva de que ornamentos são maladaptativos em fêmeas, sugerindo benefícios que são dependentes do contexto social.

## Introduction

Colorful ornaments are employed by a variety of animal species as signals of condition or sex (Cuthill et al. 2017). Ornaments can lead to evolutionary tradeoffs if higher signal strength facilitates resource acquisition (e.g., territories or mates) but also leads to fitness costs such as increased detectability by predators (Rosenthal et al. 2001; Engqvist et al. 2015). Such costs can be exacerbated if the expression of ornaments is genetically linked between males and females but one sex incurs costs from involuntary signaling (Lande 1980; Swierk and Langkilde 2013; Clark and Rankin 2020). Alternatively, ornaments in females may play a role in sexual mimicry and confer an adaptive advantage in species where females are harassed by males (Scott 1986; Muller and Wrangham 2002), or can be important in mediating intraspecific competition (LeBas 2006; Kraaijeveld et al. 2007). The ability for each sex to modulate the signaling strength of labile ornaments—for example, behaviorally—should thus allow males and females to maximize fitness.

Behavioral modulation of ornaments can provide clues to whether they yield sex- or context-specific benefits if behaviors exaggerate (Hagelin 2002; Hogan and Stoddard 2018) or mask (Steinberg et al. 2014) ornaments. For example, male and female unicornfish (*Naso sp.*) exhibit sexually monomorphic protuberances on their heads. While males display rapid color changes of these structures during courtship and competition, this is rarely seen in females (Arai and Sato 2007). Color properties of ornaments can also be influenced by abiotic factors such as temperature, humidity, and luminosity (Walton and Bennett 1993; King et al. 1994; Shawkey et al. 2011; Langkilde and Boronow 2012; Stevens 2016). Gliding lizards enhance the signaling strength of their dewlaps by positioning themselves perpendicular to the sun during displays (Klomp et al. 2017). Thus, animals may be able to use behavior to leverage abiotic factors that affect the appearance of their ornaments, potentially offsetting sex-specific costs or incurring benefits.

Some lizard species in the genus *Sceloporus* express ventral blue badges that are variable in size and color saturation within populations and are employed in social interactions (Quinn and Hews 2003; Swierk et al. 2012). In the eastern fence lizard, *S. undulatus*, the size of the blue portion of throat badges is similar across sexes, but the color is much more saturated in males (Cooper and Burns 1987; Stephenson et al. 2017; Assis et al. 2021). The structural color of badges responds strongly to ambient temperature, changing hue from green to blue and increasing overall color saturation when warmer (Langkilde and Boronow 2012; Stephenson et al. 2017). Badge area, however, is static in relation to temperature. In both sexes, a shift of 10°C makes badge color significantly more saturated in an iguanid visual model (Assis et al. 2020). Against their pale venters, this increased saturation results in an increase in contrast and, consequently, increased visibility to receivers (Endler 1992; Doucet et al. 2007). This trait plays a role in sex identification (Cooper and Burns 1987; Swierk and Langkilde 2013) and may be relevant to fitness. In males, more saturated badges potentially signal greater condition and immune response (Assis et al. 2021), and in both males and females bluer hues or larger badges are associated with increased sprinting speed (Assis et al. 2018; Robinson and Gifford 2018; but see Robinson et al. 2021). Potential fitness costs of these ornaments have been identified in females, including reduced offspring survival and reduced coloration of male offspring in adulthood (Assis et al. 2022). Thus, thermoregulatory behavior is one way in which female fence lizards might modulate badge signaling strength and doing so in the appropriate contexts could maximize fitness.

If badges in female fence lizards are socially advantageous, females with larger badge size (a static trait) should exploit this phenotype when conspecifics are present and select environments that promote higher body temperatures, enhancing the badge saturation and thus its visibility against their pale venter. The variability seen in this trait and how it is affected by environmental temperature provide an excellent opportunity to explore the role of female ornaments in social interactions. In this study, we test the hypothesis that badge size and color saturation (i.e., degree of ornamentation) in females correlates with thermoregulatory behavior, which modulates its visibility (Assis et al. 2020). Wild-caught female lizards were housed in pairs and provided opportunity to thermoregulate in a wide temperature gradient. Shared food resources in trials along with the absence of predators were employed to promote social interactions between females that could be mediated by these visual signals. We predicted that females with larger badges or badges with higher standardized saturation would attain higher body temperatures and consequently more visible ornaments in the presence of a conspecific, indicating an association between phenotype and behavior.

## Methods

Mature female lizards were captured from sites in Tennessee (*n* = 5) and eastern Arkansas (22) and transferred to an animal housing facility. Throat badges were quantified via ventral photographs (for area) and spectrophotometry (for saturation). Saturation scores were adjusted to an iguanid visual model (Macedonia et al. 2009; Assis et al. 2020), so that individual variation more closely reflects variation perceived by conspecifics. Three of the females had no obvious badges, giving us full representation of the badge size gradient seen in the species. Nevertheless, high-resolution photographs allowed miniscule color patches to be identified (i.e., ∼3 scales), which were measured and assigned a badge area. Similarly, spectrophotometry allowed us to detect reflectance on the blue spectrum from these small color patches.

Animal collection was authorized by the respective states’ permits. Animal housing and behavioral trials adhered to Guidelines for the use of Animals in Research, the legal requirements of the U.S.A., the Institutional Guidelines of The Pennsylvania State University, and were approved by the Institutional Animal Care and Use Committee (IACUC). Further information about animal sampling and housing, color quantification, and observations regarding *Sceloporus* taxonomy can be found in the *Supplementary material*.

### Thermoregulatory behavioral observations

Female lizards were housed in pairs from the same collection site and acclimated to housing conditions and human observers for a minimum of one month. All were sexually mature and non-gravid (determined via abdominal palpation). After this period, each lizard was paired to a new female from the same site in a new enclosure, and acclimated for 48 h before the start of behavioral trials. Acclimation and trials were carried out in the same type of enclosures as the home enclosures (45 × 30 × 25 cm), with two modifications. First, a cylindrical wooden perch (height: 10 cm, diameter: 5 cm) with carved grooves was positioned vertically at one end of the enclosure, and a lamp with a 60 W incandescent bulb was positioned 15 cm above the top of the wooden cylinder. The lamp was surrounded by aluminum foil, leaving an aperture of ∼5 cm diameter to limit the area of heat radiation. Second, the temperature in the room was reduced to 20°C during trials. These modifications were made so that enclosures would capture a wider temperature gradient, seen in the range of body temperatures measured (Table 1).

**Table 1:** Summary statistics for the response variable and its predictors. SD = standard deviation; Min, Max = minimal and maximal values across all trials; CV = coefficient of variation. Badge saturation is a fraction of 1, corrected for an iguanid visual system (see *Supplementary material*)

	Mean	SD	Min	Max	CV
Body temperature (°C)	27.3	2.8	22.8	36.8	0.102
Badge saturation	0.433	0.1	0.243	0.598	0.221
Badge area (cm^2^)	0.103	0.04	0.03	0.201	0.39
SVL (cm)	6.99	0.87	5.6	8.3	0.125

One hour after the heat lamps were turned on, at 0845 h, we measured the body temperature of each lizard, on two consecutive days. We prioritized this early measurement in our analysis because (1) both individuals in a pair would have been at equivalent baseline temperatures after the previous night and (2), lizards are typically more motivated to thermoregulate in the first hour of the morning (Díaz 1991; Carrascal et al. 1992). Moreover, although badges quickly change color when warming up, the reverse is not true—badge color retains its properties for considerably longer after the individual cools down to a baseline temperature (Stephenson et al. 2017). This makes early morning realized thermoregulation more informative of potential social significance of color enhancement. To avoid handling animals during trials, we measured external body temperature using a Raytek MT4 infrared thermometer (Raytek corporation, Santa Cruz, California, USA) at approximately 10 cm from a lizard's torso (distance-to-spot ratio = 8 : 1). External body temperatures obtained using this method are significantly related to cloacal temperatures of the same lizards obtained with a thermocouple (MacLeod et al. 2019), but we are unable to determine how our measurements may differ from true internal body temperatures of these individuals. Still, because the procedure was standardized across individuals, we expect variation in the data to be representative of thermoregulation differences.

After two days of temperature measurements, each lizard was paired to another lizard from the same collection site for new acclimation and trials, as sample sizes allowed. This resulted in each lizard being involved in two or three trials. By assigning each female to multiple trial pairs, we tried to account for any effect that varying conspecifics might have on a focal individual's thermoregulatory behavior (while also factoring that in statistically, see *Statistical analysis*). Coefficients of variation for snout-to-vent length (SVL) within a pair ranged from zero to 0.18.

Because of their feeding schedule, lizards had been fed either on the first or second day of temperature measurements, or during the acclimation period. Alternate feeding states have been shown to influence thermoregulation in *S. jarrovii* (Beal et al. 2014) and likewise could have influenced results seen here. However, the feeding and trial schedules also allowed alternative feeding states for each lizard to be represented in trials, albeit confounded with new pair matchings. In total, 148 temperature measurements were collected, divided among two observations per lizard in 37 trials. The arena's warmest region (top of the cylindrical perch immediately below lamp) was occupied by either lizard in a pair in only 26 out of 74 observations (35%). Therefore, we concluded that no intense competition for the warmest microhabitat occurred, and that lizards were not thermally limited under trial conditions.

### Statistical analysis

All analyses were done in R version 4.1.1 (R Core Team 2021). We used a linear mixed-effects model (Bates et al. 2015; Kuznetsova et al. 2019) with body temperature of a focal individual as the response variable. Body temperature was natural log-transformed to improve the normality of model residuals. Predictors in the full model were relative badge area, saturation, SVL, all combinations of two-way interactions, and the three-way interaction between predictors. Site of origin (*n* = 2) was included as a fixed factor. Random effects were focal individual's identity (*n* = 27) and pair identity (*n* = 37). We then used the *dredge* function in the R package *MuMIn* (Bartoń 2018) to compare AICc scores and weights (Akaike Information Criterion, corrected for small sample sizes) of all subsets of this full model (Table 2). The most parsimonious model retained relative badge area as its only predictor. Model assumptions of this and the full model were visually inspected by plotting residuals vs. fitted values, and models were inspected for highly influential data points (Cook's distance), which we did not observe.

**Table 2:** Predictors retained (X) in top candidate models based on AICc scores. *Area*: badge area relative to head area; *Saturation*: color saturation modeled for an iguanid visual system; *SVL*: snout-to-vent length; *Site*: individual's origin site (TN or AR). AICw: AIC weights

Area (A)	Saturation (S)	SVL	Site	A x S	A x SVL	S x SVL	A x S x SVL	Δ AICc	AICw
X	–	–	–	–	–	–	–	0	0.169
X	–	X	–	–	–	–	–	0.81	0.113
X	–	–	X	–	–	–	–	1.79	0.069
X	X	–	–	–	–	–	–	2.12	0.059
X	X	X	–	–	–	–	–	2.36	0.052
X	–	X	–	–	X	–	–	2.64	0.045

## Results

**Fig. 1:**
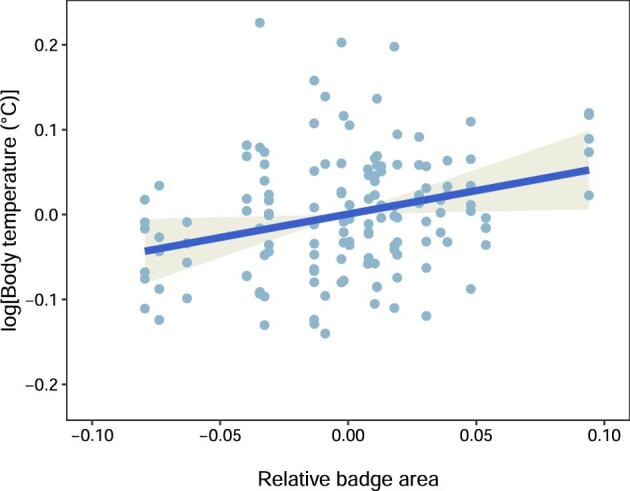
Predicted values for natural log-transformed body temperature in response to relative badge area (linear mixed-effects model: β = 0.55, df = 19.12, *P* = 0.038). Each lizard (random effect, *n* = 27) had its temperature measured twice in each of two or three different lizard pairings (random effect, *n* = 37) for a total of 148 non-independent temperature measurements.

Means, standard deviations, ranges, and coefficient of variations for body temperature, absolute badge saturation, absolute badge area, and SVL are presented in Table 1. AICc scores for top candidate models and predictors within each are shown in Table 2. The only predictor to be represented in all top models (Table 2) was female badge area. Two other models had a ΔAICc < 2, but these were more complex (had more predictors) and for this reason were disfavored. Substituting SVL in the full model for body mass or for the linear residuals of mass on SVL led to the same outcome: the top model (ΔAICc = 0) retained only female badge area. The null model (containing no predictors) had a ΔAICc = 2.65, so we performed a likelihood ratio test (LRT) between the top model and the null model. LRT results corroborated our interpretation that the model containing badge and random effects was the best predictor of realized body temperatures in our dataset (χ^2^ = 4.79, df = 1, *P* = 0.028). This model indicates that females with larger badges relative to their heads attained higher body temperatures (β = 0.55, t = 2.2, df = 19.12, *P* = 0.038; Fig. 1).

## Discussion

Our results show a relationship between phenotype and behavior in female fence lizards: females with larger blue badges (i.e., more ornamented) thermoregulated to attain higher body temperatures than females with smaller badges when all had access to a range of temperatures. Temperature differences within the range of our observations (14°C, Table 1) are known to significantly increase the saturation of male and female badges within the range predicted to be discernible under an iguanid eye model (Langkilde and Boronow 2012; Stephenson et al. 2017; Assis et al. 2020), and consequently their contrast and visibility increases against their pale ventral background. Thus, females that were more ornamented exhibited ornament-enhancing thermoregulatory behavior while in the presence of a conspecific.

Various scenarios have been observed to be, or proposed as, viable drivers for the adaptive evolution of female ornamentation. These range from competition for ecological resources (LeBas 2006; Tobias et al. 2012) to sexual selection (Rubenstein and Lovette 2009) and sexual mimicry (Gosden and Svensson 2009). In circumstances conducive to social competition (e.g., in our trials: conspecific presence, communal feeding, predator absence), it should follow that females would benefit from enhancing such signals (i.e., exhibit higher thermal preference) given the opportunity to do so. Because we focused on the behavior and phenotype of females, we cannot determine the role that male presence might have had on thermoregulatory preferences and signal enhancement (Scott 1986; Lee et al. 2019). However, exploitation of masculinized traits by females can reduce competition costs imposed by other females. Male-mimicking female spotted hyenas are less likely targets of aggression by other females, and consequently may experience reduced levels of sibling aggression, infanticide by conspecific females, and territory competition (Muller and Wrangham 2002). Therefore, it is plausible that warmer female fence lizards with more visible male-typical ornaments could experience analogous fitness advantages.

Temperature is a critical resource with important effects on the physiology and fitness of ectotherms (Huey and Berrigan 2001). Like other reptiles, fence lizards exhibit optimal energy intake, energy consumption, and performance at specific temperatures (Angilletta Jr. 2001; Angilletta Jr et al. 2002; Robinson and Gifford 2018). In our experiment, the arena's warmest microhabitat was unoccupied during 65% of observations, suggesting that individuals were not thermally limited even in a shared environment. The interaction between thermoregulation and color change is an important factor mediating communication and camouflage in ectotherms (Stuart-Fox and Moussalli 2009). In fence lizards, color sensitivity to temperature and its importance to social interactions introduce interesting variables to their thermoregulatory behavior and physiological demands, which likely are context dependent (Sinervo et al. 2010).

Sexual ornaments with incomplete sexual dimorphism are often seen as a manifestation of intralocus sexual conflict (Chapman 2006; Bonduriansky and Chenoweth 2009). If rudimentary male-typical ornaments are maladaptive to females, they should behave to reduce their conspicuousness. Contrary to that prediction, female fence lizards with larger badges maintained higher body temperatures, making color more saturated and thus likely more visible to conspecifics. This finding sheds important light on the potential adaptive significance of female ornamentation: albeit rudimentary, the visibility of female ornaments may be behaviorally manipulated in appropriate contexts, warranting further investigation on the potential social benefits of these traits.

## Supplementary Material

obac029_Supplemental_File

## Data Availability

All data are available on ScholarSphere doi.org/10.26207/w643-fm37.
