# Supplementary material for: Masculinized Sexual Ornaments in Female Lizards Correlate with Ornament-Enhancing Thermoregulatory Behavior
Source: Integr Org Biol. 2022 Aug 25;4(1):obac029. doi: 10.1093/iob/obac029 (PMC9409079; doi:10.1093/iob/obac029)
Supplement: obac029_Supplemental_File [file obac029_supplemental_file.docx]

**Masculinized sexual ornaments in female lizards correlate with ornament-enhancing thermoregulatory behavior**

Braulio A. Assis, Julian D. Avery, Ryan L. Earley, Tracy Langkilde

**Supplementary material**

**Methods**

*Animal collection and husbandry*

We captured 27 adult *Sceloporus undulatus* females (snout vent length, SVL > 56mm) from populations in Tennessee (Edgar Evins State Park, Standing Stone State Park, and Land Between the Lakes National Recreation Area) and Arkansas (Mississippi River State Park and private lands in Lee County). Some taxonomic uncertainty exists regarding *Sceloporus* populations in eastern Arkansas. Leaché (2009) modeled phylogenetic relationships for the *Sceloporus undulatus* species group in North America and provided support for the *S. consobrinus* phylogenetic groups occurring across much of Arkansas. However, sampling for that work did not include lizards from Arkansas *per se*, our collection site is found outside the *S. consobrinus* cloud modeled in that work, and *S. consobrinus* populations are predicted to extend west of the Mississippi River into Mississippi, which could indicate some gene flow between the two populations. Considering these points, we do not have overwhelming evidence to characterize the two populations as separate species and refer to all lizards in this study as *Sceloporus undulatus*. Nevertheless, our behavioral trials were carried out with pairs of lizards necessarily from the same population, and site of origin was not a significant predictor of thermoregulatory behavior in our model (see *Results*).

On the day of collection, we measured each female’s snout-to-vent length using a plastic ruler (to the nearest mm) and body mass using an electronic scale (to the nearest 0.1 g). Lizards were then brought to an animal housing facility at the Pennsylvania State University. Until the day of the trials, females were housed in pairs in a plastic enclosure (45 x 30 x 25 cm) lined with paper towels and with a 15 x 15 cm piece of opaque corrugated plastic to be used as shelter, which were all replaced after conclusion of a trial. Lizard SVL was 7 ± 0.86 cm (mean ± SD) and thus, the two females occupied on average ~2% of the enclosure’s area. A lamp with a 60 W incandescent bulb was hung 25 cm above one end of the container and turned on daily from 0800 to 1600 hours to provide heat for basking. Pairs of females were fed five to six adult *Acheta domesticus* crickets three times per week, with one of these feeds supplemented with Reptivite™ reptile vitamins (Zoo Med Laboratories Inc., San Luis Obispo, CA, USA). A small dish containing water was available to animals at all times and the photoperiod was maintained at 12 h light:12 h dark. Temperature in the room was maintained at ~23°C, considerably below their preferred body temperature of ~34°C (Angilletta Jr et al. 2002), in order to create a wider temperature gradient in the enclosures.

## Color quantification

We used an Ocean Optics Jaz UV/VIS spectrometer with a pulsed xenon light source to measure reflectance of female fence lizard badges. The light source was held perpendicular to the subject and spectra were calculated relative to a diffuse white standard (Ocean Optics WS-1) using SpectraSuite (2006). The probe was enveloped by black tubing that blocked ambient light and allowed us to place it directly on the animal at a standardized distance. Prior to measurements, the spectrometer was calibrated for ambient interference by placing the probe into a lightless black box. We measured reflectance of the colored portion of the lizard’s left throat badge with an integration time of 40 µs and a trigger period of 10 µs. We measured each individual lizard’s badge color three times, removing and replacing the probe each time within the colored region. For each measurement, SpectraSuite averaged 5 scans. We then used the R package *pavo* (Maia et al. 2019) to calculate color saturation as perceived by conspecifics by fitting spectral data to a visual sensitivity model. Sensitivities for the four cone types in *Sceloporus undulatus* are not yet determined, so instead we used the parameters established for the closest related iguanid for which these are available, *Crotaphytus dickersonae* (Crotaphytidae) – 359nm, 459nm, 481nm, 558nm (Macedonia et al. 2009) – while assuming even ratios of cone types. Saturation scores (**r**) were projected in a tetrahedral space and corrected for the maximum **r** length possible for that hue in a non-spherical space (Stoddard and Prum 2008; Maia et al. 2019). Finally, because color saturation in *S. undulatus* is positively associated with body temperature (Langkilde and Boronow 2012; Stephenson et al. 2017; Assis et al. 2020), we standardized saturation by extracting the residuals of saturation linearly regressed on body temperature at the time of measurement. Temperature at measurement was uncorrelated with saturation (β = 0.01, df = 25, p = 0.36). This is likely due to the relatively small range of body temperatures these lizards exhibited at testing (32.2 to 37.8°C; mean ± SD: 35.2 ± 1.6).

We used ventral photographs of females to estimate the area of throat badges. Females were placed on their backs and had two photographs taken from directly above, with a plastic ruler in the frame as a scale. The areas of the right throat badges were measured in ImageJ using the Polygon tool, averaged from two distinct photos per individual. The same procedure was used to measure the ventral surface area of the head of all individuals, where badges are located, so that badge area could be calculated relative to head area. This allowed us to determine the proportion of the available ventral surface of the head that was occupied by these ornaments. This relative badge area was calculated by extracting the residuals of a linear regression of badge area on head area. Body size (mass) affects rates of thermoregulation, and was measured and included in our model (see *Results*). The repeatability of badge area and head area measurements was tested using the R package *rptR* (Stoffel et al. 2017), based on a normal distribution and 1000 parametric bootstraps. Both measurements were highly repeatable. For badge area: R = 0.869, 95% confidence interval (CI) = [0.721, 0.935], p < 0.0001. For head area: R = 0.958, CI = [0.909, 0.981], p < 0.0001.

**References**

Angilletta Jr MJ, Hill T, Robson MA. 2002. Is physiological performance optimized by thermoregulatory behavior?: a case study of the eastern fence lizard, *Sceloporus undulatus*. J Therm Biol. 27:199–204.

Assis BA, Jarrett BJM, Koscky G, Langkilde T, Avery JD. 2020. Plastic sexual ornaments: assessing temperature effects on color metrics in a color-changing reptile. PLoS One. 15:e0233221. doi:10.1371/journal.pone.0233221.

Langkilde T, Boronow KE. 2012. Hot boys are blue: temperature-dependent color change in male eastern fence lizards. J Herpetol. 46:461–465. doi:10.1670/11-292.

Leaché AD. 2009. Species tree discordance traces to phylogeographic clade boundaries in North American fence lizards (*Sceloporus*). Syst Biol. 58:547–559. doi:10.1093/sysbio/syp057.

Macedonia JM, Lappin AK, Loew ER, Mcguire JA, Hamilton PS, Plasman M, Brandt Y, Lemos-Espinal JA, Kemp DJ. 2009. Conspicuousness of Dickerson’s collared lizard (*Crotaphytus dickersonae*) through the eyes of conspecifics and predators. Biol J Linn Soc. 97:749–765.

Maia R, Gruson H, Endler JA, White TE. 2019. pavo 2: new tools for the spectral and spatial analysis of colour in r. Methods Ecol Evol. 10:1097–1107. doi:10.1111/2041-210X.13174.

Stephenson BP, Ihász N, Byrd DC, Swierk J, Swierk L. 2017. Temperature-dependent colour change is a function of sex and directionality of temperature shift in the eastern fence lizard (*Sceloporus undulatus*). Biol J Linn Soc. 120:396–409. doi:10.1111/bij.12870.

Stoddard MC, Prum RO. 2008. Evolution of avian plumage color in a tetrahedral color space: a phylogenetic analysis of new world buntings. Am Nat. 171:755–776. doi:10.1086/587526.

Stoffel MA, Nakagawa S, Schielzeth H. 2017. rptR: repeatability estimation and variance decomposition by generalized linear mixed-effects models. Methods Ecol Evol. 8:1639–1644. doi:10.1111/2041-210X.12797.
